# A cost-effective 200 kV cryo-EM core facility for high resolution single particle analysis

**DOI:** 10.1515/mim-2025-0039

**Published:** 2026-04-06

**Authors:** Colin C. Gauvin, J. Luke Findlay, Coltran Hophan-Nichols, C. Martin Lawrence

**Affiliations:** Department of Chemistry and Biochemistry, 33052 Montana State University, Bozeman, MT, USA; Research Cyberinfrastructure, 33052 Montana State University, Bozeman, MT, USA

**Keywords:** Talos, Arctica, Gatan, K3, SerialEM

## Abstract

We sought to establish a sustainable 200 kV cryo-EM core facility for the Northern Rocky Mountain region with high resolution single particle capabilities. With modest funding, relative to 300 kV instruemnts, from the NSF, the Murdock Charitable Trust and Montana State University, we remodeled space and installed a 200 kV Talos Arctica with a Gatan K3 camera, along with supporting software and computational resources.  In addition, a refurbished T-12 Spirit was added to support negative stain work, and the instrumentation was collectively organized as a university core facility. The facility is now serving academic and industrial users across the Northern Rocky Mountain region, and beyond. We find that single particle data collection on low symmetry particles greater than 100 kDa routinely gives structures at better than 3 Å resolution, with several high symmetry structures going past 2 Å. These efforts demonstrate that Carnegie R1 - research intensive universities are well served by 200 kV microscopes for single particle work. Such microscopes approach the capabilities of more expensive 300 kV instruments with their ability to produce high resolution single particle structures, and can do so at significant cost savings, contributing to sustainable core facilities.

## 1 Introduction

Structural biology has had an outsized impact on our understanding of biology at the molecular level. An early example is the crystallization of urease by Sumner in 1926, which showed that enzymes were proteins that could be isolated in pure form while retaining enzymatic activity [1]. This was followed by high resolution 3D structures for myoglobin, hemoglobin, insulin, double stranded DNA, viruses, the photosynthetic reaction center, ion channels, the F_o_F_1_ ATP synthase, ribosomes, G-protein coupled receptors, and many more. Today, a century after Sumner’s seminal work, there are more than 245,000 experimentally determined structures in the Protein Data Bank. Collectively, these structures have revolutionized our understanding of the molecular basis of life. And in today’s world, they are also fundamental for training AlphaFold and its successors. Along the way, the experimental techniques used to determine macromolecular structure have expanded from X-ray diffraction to include NMR and cryo-EM single particle analysis. And with the “resolution revolution”, cryo-EM has become the preferred method for structural analysis of large macromolecular complexes.

However, the cost of 300 kV transmission electron microscopes and direct electron detectors is beyond the means of many universities and research institutes. For this reason, significant efforts have been made to “democratize” cryo-EM [2]. In the United States, this includes the establishment of the NIH Common Fund Transformative High Resolution Cryo-Electron Microscopy Program Centers (cryoemcenters.org), providing access to state-of-the-art equipment. On the other end of the spectrum, manufacturers have worked to reduce instrumentation costs, developing highly capable 200 kV and even 100 kV transmission electron microscopes, along with direct electron detectors optimized for these energies. Critically, Herzik, Lander and others have demonstrated the ability of 200 kV microscopes and direct electron detectors to provide high resolution structures, even on sub 100 kDa particles [3], [4], [5], [6], [7], [8], [9]. This represents an important paradigm shift for the design of cryo-EM core facilities. Specifically, high resolution single particle structures can be done with lower cost 200 kV instrumentation, with access to equipment at the National Centers when needed. With this goal in mind, we undertook the establishment of a cryo-EM core capable of high resolution single particle analysis at Montana State University, whose mission is to serve a five state region (Montana, Eastern Washington, Idaho, Wyoming and North Dakota) in the Northern Rocky Mountains and Northwestern Plains of the United States.

## 2 Materials and methods

### 2.1 Site preparation

**Figure 1:**
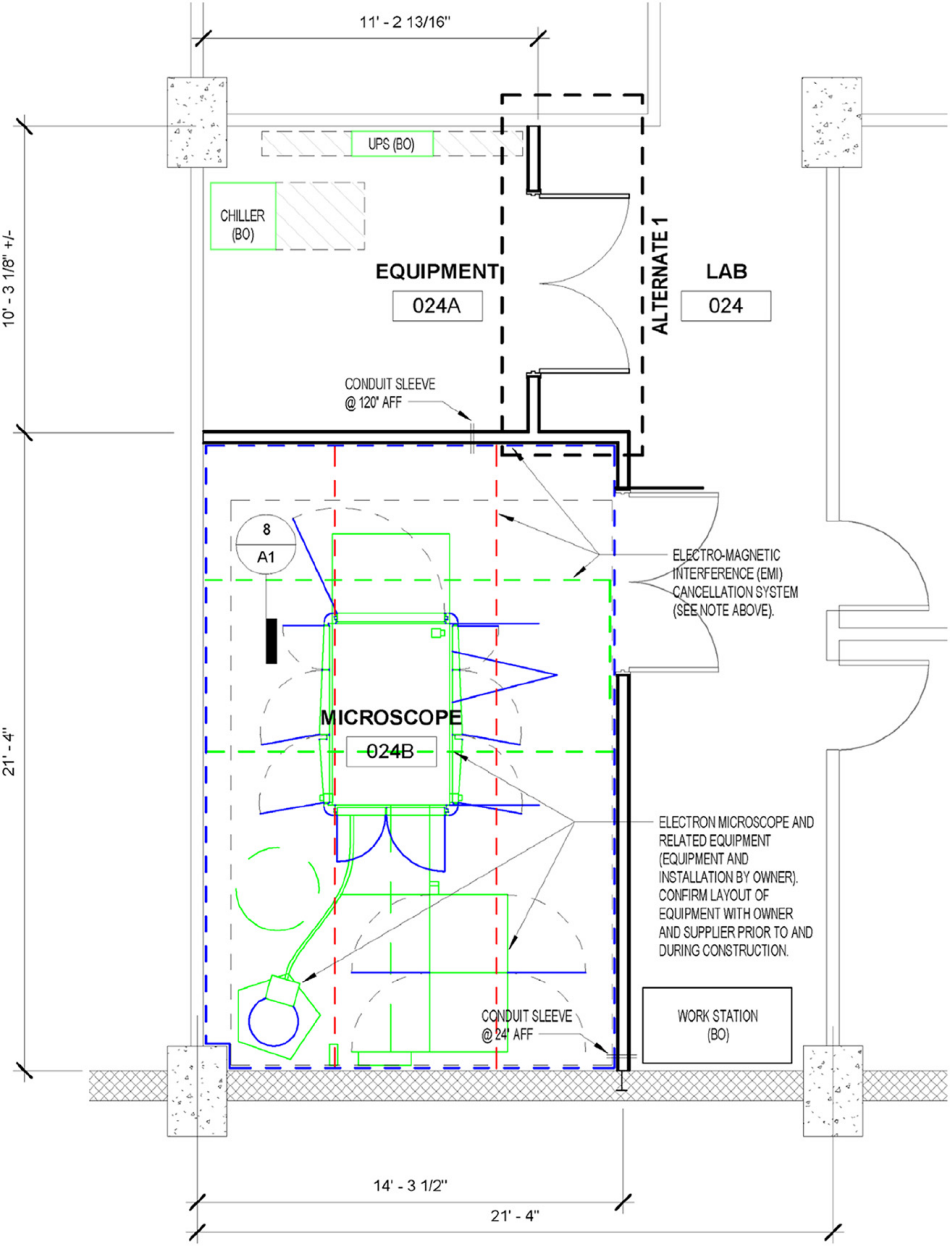
Microscope and equipment room design. Following demolition, the room space was partitioned into 3 rooms; one for the MICROSCOPE, an EQUIPMENT room to house the water chiller, Gatan server and storage, and the LAB, to house workstations for microscope control, the plasma cleaner, vitrobot, clipping station and other peripheral equipment. The partial room at the top of the figure is not part of the facility, it connects to the neighboring mass spectrometry core. The location of the Arctica, high tension tank and associated electronics cabinets are indicated in solid green lines, with cabinet doors in solid blue. The cable locations for the Spicer EMI cancellation system are shown as dashed lines. The dimensions of each room are indicated, for example the MICROSCOPE room is 14 feet 3½ inches by 21 feet 4 inches.

Site selection and remodeling to accommodate the Talos Arctica are described in Results, with a few details on several specific aspects provided here:

*EMI –* To minimize AC EMI, all original HVAC, conduit and lighting were removed from the microscope space, with all reconstructed controls and valves relocated outside the room (Figure 1). Newly installed electrical was restricted to essential microscope and camera connections, peripheral outlets, and LED lighting. An active EMI cancellation system was integrated by Spicer, featuring 6 mm cables installed in shallow floor grooves beneath anti-static flooring and supported overhead by ceiling-mounted unistruts with 4–5 inches of clearance between the cables and any parallel supports. The vertical Spicer cable runs avoided power rough-ins and doorways to allow unobstructed roll-in access for LN2 Dewars (Figure 1).

*Acoustic –* The remodel utilized SoundBreak XP acoustic wall board.

*Thermal Stability –* Temperature control utilized the building HVAC supply piped in through a set of 8 air diffusers that ring the upper perimeter of the microscope room, and a single return 3 feet above the floor, with a thermostated sensor just inside the return grille. The thermostat was set to 68 °F and the deadband on the thermostat was reset to 1 °F Δ*T*. Overall, with supply air flow at 1,100 CFM, the room is under positive pressure with minimal temperature fluctuation, even when room doors are temporarily open.

*Data Transfer –* Eight 10 GbE ports to facilitate data transfer from the camera server to local secure storage (Blackmore) and off campus destinations were located adjacent to the camera server in the equipment room.

### 2.2 Equipment

The following equipment choices were made for the core facility.

#### 2.2.1 Talos Arctica

Final decisions on specific equipment and configurations were made in early 2019. Ultimately, we chose proven technology whenever possible. Thus, of several excellent 200 kV microscopes available at that time (CRYO ARM 200, Talos Arctica, Glacios), we were drawn to the Talos Arctica. In addition, we wanted to preserve the option to add an energy filter down the road, which was not an option for the Glacios at that point (ThermoFisher had not yet announced their Selectris energy filters). Due to the height of our existing doorways, we opted for the reduced Talos ship height. Additional options that might have been considered for the Arctica at the time of purchase include the addition of a smaller beam stop and aperture needed for micro-crystal electron diffraction (MicroED), and perhaps substitution of the Ceta-D camera for the Ceta-M, especially if the facility is seriously considering a significant volume of MicroED work.

#### 2.2.2 Gatan K3 direct electron detector

With regard to cameras, the earlier successes of the Gatan K2, along with the anticipated DQE, high frame rate, and number of active pixels guided our choice of the Gatan K3 direct electron detector over the Falcon 3. Further, in June of 2018, ThermoFisher announced it would acquire Gatan. While acquisition was later canceled, at the time this suggested the K3 camera would soon be supported by EPU data collection software, effectively resulting in a single vendor solution.

#### 2.2.3 Spicer active EMI cancellation system

While electromagnetic interference (EMI) was marginally within specifications following the remodel, we elected to address residual EMI with installation of a Spicer Consulting SC24 Field Cancelling System. This specific choice was at the recommendation of ThermoFisher, and was conveniently included within the ThermoFisher quote.

#### 2.2.4 T-12 Spirit

In addition to the cryogenic side of the facility, we wanted to address the need for room temperature, negative stain capabilities. With the help of ThermoFisher, we purchased a used Tecnai G2 Spirit Twin and UltraScan 1000XP that was delivered and installed by ThermoFisher.

#### 2.2.5 Service contracts

In addition to the one year warranty, 3 years of service contract were also purchased up front for the Talos Arctica, Gatan K3 camera and T-12 Spirit.

#### 2.2.6 Other

Grant resources were also used for the purchase of EPU and Amira licenses, a Vitrobot Mark IV plunge freezing robot, NanoSoft autogrid tweezers, capsules, cassettes, loading and clipping stations, clipping tools, a Pelco easiGlow glow discharge cleaning system and modest amounts of consumables.

### 2.3 Computational infrastructure

#### 2.3.1 Hardware

##### 2.3.1.1 Microscope servers

**Figure 2:**
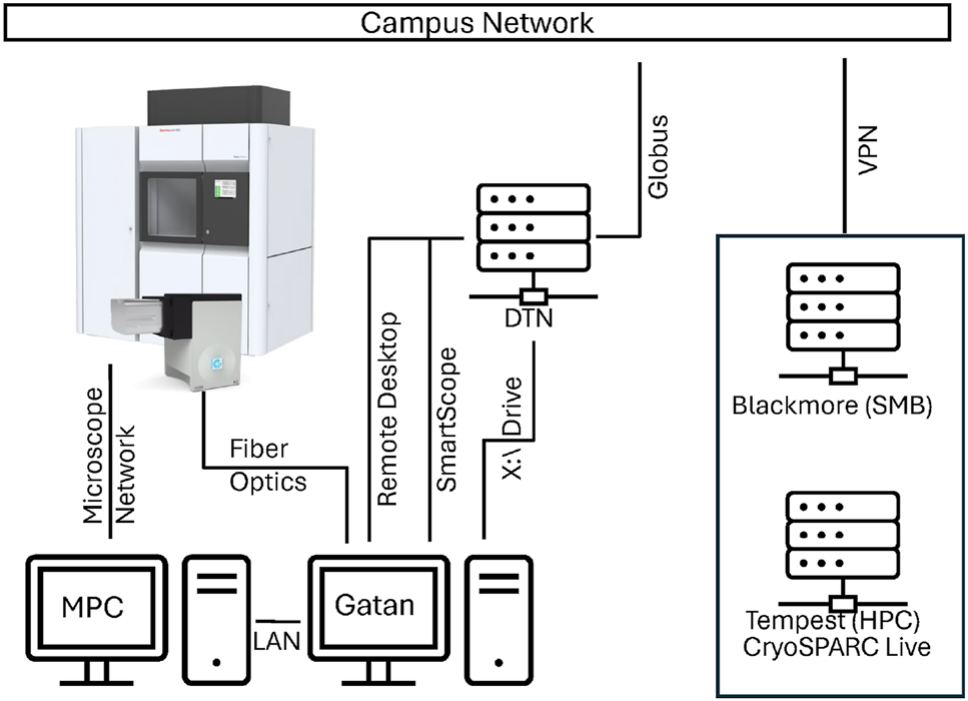
Connectivity of EM facility. The Gatan K3 server acts as the heart of data collection, containing the SerialEM installation, and direct network connections to the MPC, the camera, and the DTN. The DTN serves as the single point of ingress and egress for the network, proxying remote desktop, providing a user interface for SmartScope, and exposing the K3 server X:\ drive to Globus for data transfer. HPC and network storage, in the form of Tempest and Blackmore respectively, are available in an isolated network, accessible via the campus VPN.

The Talos Arctica came equipped with a “Microscope PC” running Windows 10, and software version 2.4.1. SerialEM’s SEM-Server was installed for the SerialEM connection [10]. Additionally, a direct network connection to a Thermo Fisher Support PC running RAPID was enabled for remote service. An additional direct network connection to the Gatan K3 server was added, and Ultra VNC Server was installed on the microscope PC to enable local network remote access from the K3 server, independent of RAPID (Figure 2).

##### 2.3.1.2 Camera servers (GMS)

The Gatan-provided camera server (Figure 2) was originally a 5-card model, running GMS 3.6.2 to which a 4-port 10 GbE network interface controller (NIC) was added. This 10 GbE connection was used to network to a data transfer node (DTN) running Globus (more details below). Additionally, a 10 GbE connection was made to the MPC for Digital Micrograph and SerialEM access. Finally, the stock display card, an Nvidia Quadro P600 was replaced with a single-slot Nvidia Quadro A4000 for faster GPU-accelerated motion correction within SerialEM. SerialEM 4.2 was installed (more details below). The RAID 0 X:\ drive that is typically used for writing movies during data collection, is exposed via SMB. While the camera server is not directly networked to the campus network or internet, it does run remote desktop software which is accessible through a proxy. Additionally, UltraVNC Viewer is installed to facilitate local control of the MPC.

##### 2.3.1.3 Research Cyberinfrastructure

Research Cyberinfrastructure (RCI; RRID:SCR_026229) is a mission critical partner for the Cryo-EM Core Facility. RCI serves as a central resource for the Information Technology (IT) and Cyberinfrastructure (CI) systems, services, and support needed to enable and promote advanced research across disciplines at Montana State University. They provide centralized support, systems, and services for IT specific to research, including high-performance computing (HPC), large scale storage, and high-performance networking and data transfer.

##### 2.3.1.4 Tempest

Tempest (Figure 2) is a subscription based high performance research cluster with a free internal access tier, administered by campus research computing (RCI) at Montana State University. A suite of commonly used cryo-EM related applications is installed and maintained on Tempest (and directly connected dedicated systems) for the local cryo-EM community. The Cryo-EM Core Facility began with an allotment of 4 A40 GPUs, but has since expanded beyond this initial purchase. So too has Tempest, which is currently comprised of 13,416 logical CPU cores, 56 GPUs, 57.35 TB of memory, and 800 TB of all-flash storage. A more detailed description of the cluster can be found at www.montana.edu/uit/rci/tempest/.

##### 2.3.1.5 Blackmore

Blackmore (Figure 2) is a subscription based, large-scale, high performance, secure storage system that, like Tempest, is also administered by RCI. Additional details can be found at https://www.montana.edu/uit/rci/blackmore/. The Cryo-EM Core Facility currently maintains 320 TB of bulk data storage. Individual laboratories maintain their own data storage subscriptions to accommodate their needs. Blackmore storage is available over SMB by access with a user’s domain credentials. A facility account was created that is used for common/shared file systems. Blackmore also integrates with Tempest as a bulk data access storage tier. While Blackmore is utilized for bulk data storage, particles stacks and other data are routinely cached on Tempest’s all-flash storage during calculations.

##### 2.3.1.6 Data transfer node (DTN)

A Dell PowerEdge R340 was installed in the facility to serve as a data transfer node (DTN, Figure 2). This DTN serves as a universal point of ingress and egress to the microscope network. It is connected to the main campus network, and thus internet, via a 10 GbE connection. Additionally, it has a direct 10 GbE connection to the Gatan K3 camera server. The DTN mounts the X:\ drive of the Gatan K3 camera server using SMB. Additionally, the DTN mounts the facility’s bulk networked storage share (Blackmore), as well as the bulk networked storage shares of frequent user groups, both academic and commercial. Globus Connect Personal (GCP) was installed as a system service (systemd) that auto-starts and exposes the mounted filesystems to a facility Globus account. The DTN also serves as a point of access from the internet, running a proxy service that drops all traffic except remote desktop traffic to the Gatan K3 camera server. This enables the camera computer to be remotely addressable without direct internet exposure. Finally, the DTN was also a convenient place to run SmartScope, accessible only over the campus network, as the camera computer X:\ drive and bulk network storage were both already mounted.

##### 2.3.1.7 Connectivity

Connectivity is a challenge for a state-of-the-art microscopy facility. Many pieces of required software are only validated against specific Windows builds, which may be out of date and missing required security patches. This can make them especially susceptible to malicious attacks over the internet, or through local networks. However, this vulnerability must be carefully balanced against the need for remote access during lengthy data collections or for remote collaborators or commercial customers, as well as the necessity to maintain high speed data off-loading connections for the many terabytes of files that may comprise a single data collection. Modern data collections typically encompass rapid screening of multiple grids, at which point data must be processed in near-real time to decide which samples should be used for extended data collection. Thus, the networking configuration must thread the needle of secure, but accessible and functional.

In our experience, a single point of entry and exit to the system dramatically simplifies the networking complexity, and also serves as a convenient point of access. While a dedicated sub-network and network switch may provide the cleanest solution, 10 GbE network switches in an enterprise environment are often accompanied by recurrent software license fees that run in the thousands of dollars. For this reason, we decided on the DTN, a Dell PowerEdge R340 running Rocky Linux and a squid proxy, with multiple 10 GbE connections as the “firewall” to the microscope network (Figure 2). Then, direct network connections between the DTN and other necessary instruments prevent any unapproved external network traffic from reaching instrument computers. Additionally, the DTN also serves as a single point of aggregation for access to files, data collection software, with a Linux operating system to run scripts or other auxiliary tools. In our network configuration, the DTN is directly networked via 10 GbE to the Gatan K3 camera server, running SerialEM. In turn, the camera server is directly networked to both the camera and the microscope PC. Finally, the microscope PC has a separate network connection to a Thermo Fisher Support PC. This support PC is connected to RAPID for remote support, but runs an up-to-date version of Windows 11 and is therefore not an imminent security concern. Remote access for collaborators and commercial clients is managed by use of a squid proxy, that accepts traffic from the internet, but drops all non-remote desktop traffic, as defined by IP address. This proxy then forwards approved remote-desktop traffic to the Gatan K3 camera server. The facility manager generates a one-time password (OTP) and communicates that to the remote user, who can use that OTP to access the camera server for the duration of their session. At the end of their session, the facility manager resets the OTP, ending their access. No other internet or campus traffic is permitted to the camera server, and all file transfers are done via direct SMB mount to the DTN.

#### 2.3.2 Software

##### 2.3.2.1 SerialEM

Data collection is done with SerialEM [10], [11], as ThermoFisher does not support EPU for Gatan K3 cameras lacking the Biocontinuum Imaging Filter (i.e., energy filter). SerialEM was installed remotely on the Gatan K3 camera server and T-12 Spirit by Gunter Resch of Nexperion (nexperion.net). The SerialEM configuration is relatively standard for single particle workflows on a two-condenser system. However, we currently use the newer image shift settling times as detailed in the SerialEM documentation, the literature [12], and below. Specifically, for multishot we routinely use a 5 × 5 pattern on R1.2/1.3 grids with approximately 7 μm of image shift from center to corner. By default, with no entries in the properties file and no advanced scripting interface, SerialEM allows 1 s of settling time per micron of image shift. This results in excessive settling times for larger shifts. We recommend enabling advanced scripting, or manually updating to the newer image shift settling times matrix:

ImageShiftDelays 4

0.1 0.0

0.3 0.4

0.5 0.5

1.0 0.5

For image shifts beyond 1 μm, SerialEM simply extrapolates. We have not found any noticeable performance degradation with these settling times, as evidenced by multiple sub-2 Å reconstructions.

Data collection rates are further enhanced by using the same spot size in View mode, as we do for Focus and Record (nanoprobe, 4). In other words, rather than reducing spot size to reduce the dose in View, we instead spread the beam. By avoiding these spot size switches during data collection, we also avoid the need to renormalize every multishot group. Finally, the “Early Returns for K2/K3” option is set to “Early return on all shots”, which enables image shift when the exposure is complete, without waiting for all frames to be written to disk before the next image shift. Importantly, we rely on an annual subscription for SerialEM support (Nexperion), which at the same time also funds continued SerialEM development. Finally, in addition to running the coma free alignment scripts in SerialEM, for multishot data collection we also rely on the coma versus image shift alignments with SerialEM prior to launching any data collection.

##### 2.3.2.2 SmartScope

SmartScope [13], which works in concert with SerialEM to further automate specimen screening and data collection through a web-based interface, was installed in consultation with Jonathan Bouvette. SmartScope is installed on the facility DTN and has access to the mounted shared storage as well as the K3 server X:\ drive. During data collection, SmartScope reads incoming data from the X:\ drive, and writes aligned images to the X:\ drive or networked storage. SmartScope further automates grid screening, and once data collection begins, speeds with SmartScope often approach 500 images-per-hour with R 1.2/1.3 grids.

##### 2.3.2.3 AnyDesk

AnyDesk remote access and support software was installed to allow authorized users to remotely monitor and control data collection.

##### 2.3.2.4 Globus

Globus [14], [15] has emerged as the *de facto* standard for transferring large cryo-EM datasets. It consists of two major pieces: Globus Connect Server, the licensed server implementation, as well as the free Globus Connect Personal, which is available as easy-to-run portable binaries that expose a single endpoint. While Montana State University maintains a Globus Connect Server license that is integrated with domain authentication, the cryo-EM facility itself egresses most of its data with a single GCP instance running on the DTN. This instance is run under a local Linux service account (called cryoem) as a system service (systemd) that auto-starts when the DTN is started. Both the Gatan K3 camera server X:\ drive, as well as the facility’s bulk networked storage are mounted as SMB shares. The facility can then use the Globus web UI to manage access to these directories for other domain accounts of collaborators and clients. Once access is initially setup, users can see both the X:\ drive and their networked storage in the web-UI, and setup single transfers, or timed synchronizations, with just a few clicks. In our experience, the rate of transfer from the X:\ drive to the networked storage, over a single 10 GbE connection with GCP on one end and GCS on the other, typically keeps pace with or exceeds our peak data collection rates.

##### 2.3.2.5 CryoSPARC

CryoSPARC [16], [17], [18], [19], [20] (including CryoSPARC live) was installed (and now regularly updated) on Tempest, the University high performance compute cluster, by Kenny Hanson (MSU Research Cyberinfrastructure) and Colin Gauvin. Different SLURM queues are available to users depending on their research group’s allocation. The CryoSPARC administration UI enables setting the visibility of each queue to a set of specific users. A queue with 4 Nvidia A40 GPUs dedicated to CryoSPARC live is available to all users during their data collection to facilitate data analysis on the fly. Once data collection is complete, individual labs then access the same CryoSPARC installation, but utilize their own and/or the free “unsafe” tempest resources to complete data analysis.

##### 2.3.2.6 RELION

RELION [21], [22], [23] is also installed and maintained on Tempest by RCI for use by the local cryo-EM community. As for CryoSPARC, the facilitie’s Tempest queues are also available to RELION users during active data collection.

### 2.4 Data collection

#### 2.4.1 Arctica specific considerations

The Talos Arctica (and Glacios) uses a 2 condenser lens systems, as opposed to the 3 and 4 condenser lens systems present in the Krios and CRYO ARM microscopes, respectively. For two condenser lens systems, parallel illumination (uniform defocus) of the specimen is limited to a single beam intensity (C2) for a given gun lens setting and spot size. High resolution work on the Talos Arctica thus requires careful alignment of the microscope to identify the optimal C2 intensity as a function of gun lens and spot size settings to achieve parallel illumination. An excellent protocol for this procedure provided by Herzik and coworkers [3], [24] is used for this purpose prior to single particle work.

In addition, to maximize camera performance and K3 camera data collection rates in counting mode, we target a flux rate of 15 e^−^ pixel^−1^ s^−1^. This is accomplished by first adjusting the guns lens settings (C1, spot size) at the desired magnification, followed by optimizing C2 for parallel illumination as described above. However, dialing through integral C1 (spot size) settings may not give the most optimal flux rate; for example, it may force a choice between an excessive rate of 17–18 e^−^ pixel^−1^ s^−1^ rate, or a sub-maximal rate of 13–14 e^−^ pixel^−1^ s^−1^. To address this, facility managers can enable the gun lens fine controls and adjust the gun lens value (from 4 to 3.7, for example) to modulate intensity at a given spot size. Notably, this will result in slight changes to the C2 value for parallel illumination, which will need to be re-optimized. On our Arctica with a pixel size of 0.872 Å, this results in an exposure time of approximately 2 s for a total dose of 40 e^−^/Å^2^ per exposure.

#### 2.4.2 Multi-shot data collection

Multi-shot, or beam image-shift data collection strategies [25], [26] were implemented with SerialEM [10] soon after microscope commissioning. More recently, SmartScope working in concert with SerialEM is used to further automate screening and data acquisition [13]. Synchronized data transfer is then used to transfer data from the Gatan server to bulk data storage on Blackmore, with subsequent on the fly data analysis using CryoSPARC live. Typical data collection rates currently range from 200 to 400+ movies per hour (5,000–10,000 movies per 24 h) in hardware binned counting mode.

### 2.5 University core facility status and RRID

Like most research universities, Montana State University maintains core-level facilities (https://www.montana.edu/research/corefacilities/red-cf.html) to house instrumentation and expertise that are shared with the campus community, external academics and industry. Working with the Office for Research and Economic Development, the Cryo-EM Facility was established as a university core, qualifying the facility for operational support from the university. As a Montana State University Core Facility, the cryo-EM facility was assigned Research Resource Identifier SCR_026324 (RRID:SCR_026324), providing a precise way to cite the Cryo-EM Core Facility. Similarly, the Research Cyberinfrastructure Facility is assigned RRID SCR: 026229.

## 3 Results

### 3.1 Funding and purchase

Funding for a multi-user cryogenic transmission electron microscope core facility for the cellular and molecular life sciences community in the northern rocky mountain region was provided by a Track II Major Research Instrumentation grant from the National Science Foundation (NSF), with required matching funds provided by the M.J. Murdock Charitable Trust and the Office of Research and Economic Development at Montana State University. The total budget, excluding space renovations, was $3,460,000. Orders were placed for a 200 kV Talos Arctica, Gatan K3 camera, Vitrobot Mark IV, a vintage T-12 Spirit and additional peripheral equipment and supplies in 2019. This included 3 years of service contract, up-front, for the Arctica, K3 and Spirit. Overall, specific instrument choices were ultimately driven by the availability of proven technology at that time. Additional details are provided in Section 2.2.

### 3.2 Design and installation

The ground floor of the Chemistry Biochemistry Building at Montana State University was constructed with isolated foundations to reduce vibration, making it a good choice for the new TEM. To further accommodate the installation, the macromolecular crystallography core was downsized and relocated, allowing the vacated space to be partitioned into a dedicated microscope room (14′ × 21′), an equipment room for the water chiller and Gatan server (10′ × 11′), and a separate control room (Figure 1).

The facility design prioritized the reduction of thermal and acoustic noise, electromagnetic interference (EMI) and vibration in order to optimize microscope performance [27]. Towards this goal, ThermoFisher provided site surveys both before and after the remodel. While initial assessments confirmed physical vibration was within acceptable limits, they identified significant EMI challenges. Notably, these were AC, not DC interference issues, and accordingly were not due to the shielded NMR magnets in the adjacent laboratory.

University engineering and architecture teams integrated the results of the site survey and pre-installation requirements provided by ThermoFisher and Gatan to ensure the new facility met all technical specifications. To minimize EMI, all original HVAC, conduit, and lighting were removed from the microscope space, and all reconstructed controls and valves were relocated outside the room. To address residual EMI following renovation, an active magnetic field cancelling system from Spicer was also installed. Additional details on the EMI, thermal and acoustic noise solutions are described in Methods. In addition, a remote transformer and power conditioner were sited in a distant building electrical room, an oxygen sensor and specialized ducting for SF6 gas exhaust were installed within the microscope room, and 10 GbE ports were brought into the equipment room. The design also benefited from established building resources, including house chilled water, compressed air, nitrogen gas, and a bulk LN2 storage dewar and filling station. Additional details are available on request. The renovation work was done in 2020, during the height of the Covid pandemic, delaying equipment installation to early 2021.

### 3.3 Commissioning

ThermoFisher provided mouse apoferritin samples for the initial workflow validation. 936 movies were collected with the parameters provided in Table 1. Initial processing by ThermoFisher with cisTEM [28] used 83 images with CTF fits better than 4.0 Å and 6,852 particles. Following 2D classification and *ab initio* 3D reconstruction, 3D Auto Refine gave a map at 3.32 Å by GSFSC. Subsequent reprocessing in our hands (CCG) with CryoSPARC utilized 102,003 final particles and gave a 2.44 Å map (Nyquist @ 2.3 Å), suggesting the newly installed microscope was performing well, that we were ready to address a sizable queue of potential projects, and that smaller pixel sizes should also be considered.

**Table 1:** Exemplar maps.

	Mouse apoferritin	DPSL/mini-ferritin	50S ribosome	HmuS dechelatase
PDB ID	NA	PDB 9CZ0	PDB 9YPH	PDB 9D26
EMDB ID	NA	EMD-46055	EMD-73300	EMD-46483
	508 kDa	258 kDa	1.47 MDa	163 kDa
Magnification	45,248	72,463	55,187	55,187
Voltage	200 kV	200 kV	200 kV	200 kV
Exposure rate	15 e^−^/pix/s	15 e^−^/pix/s	13.2 e^−^/pix/s	18.3 e^−^/pix/s
Total exposure	50 e^−^/Å^2^	55 e^−^/Å^2^	54 e^−^/Å^2^	56 e^−^/Å^2^
Number of fractions	50	35	50	51
Defocus range	−0.8 to −2.0 µm	−0.6 to −1.5 µm	−0.5 to −2.5 µm	−0.6 to −1.5 µm
Pixel size	1.152 Å	0.345 Å (super-res)	0.552 Å (super-res)	0.906 Å
Software	cisTEM/CryoSPARC	CryoSPARC	CryoSPARC	CryoSPARC
Initial number particles	6,852/unknown	3,569,482	1,665,000	18,955,798 (blobs)
Final number particles	6,852/102,003	2,583,454	642,285	2,133,842
Symmetry imposed	432 (O)	23 (T)	C1	C1
GSFSC resolution (masked)	3.32 Å/2.44 Å	1.86 Å	2.25 Å	2.60 Å
B-factor	98.2 Å^2^	52.7 Å^2^	63 Å^2^	120.8 Å^2^

### 3.4 Subsequent operations

#### 3.4.1 Single particle analysis

We thus embarked on several single particle projects as we explored the capabilities of our specific instrument.

##### 3.4.1.1 DPSL mini-ferritin

**Figure 3:**
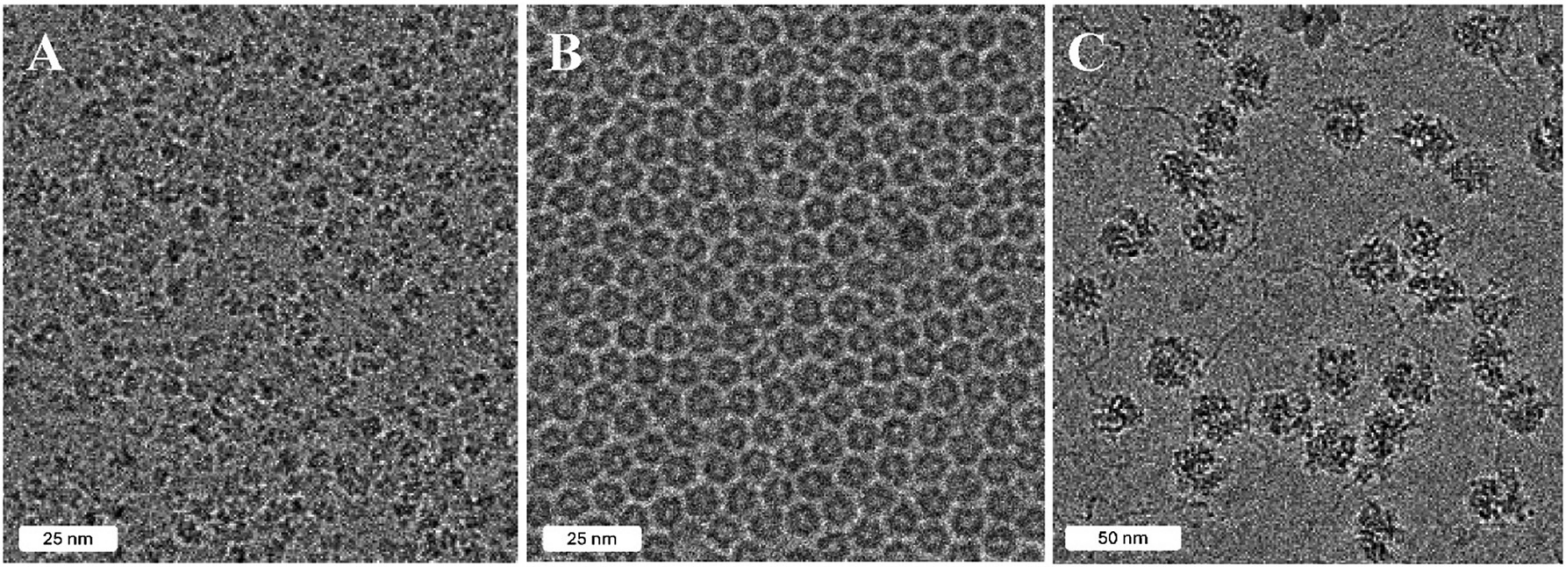
Representative micrographs. Selected areas of representative micrographs are shown for (A) Bacteroidetes thetaiotaomicron HmuS at 1.5 mg/mL, −1.19 µm defocus, (B) *Pyrococcus furiosus* DPS-like protein at 2 mg/mL, −1.08 µm defocus, and (C) 50S ribosomal subunits from *Pseudomonas aeruginosa* at 2.3 mg/mL, −1.41 µm defocus.

**Figure 4:**
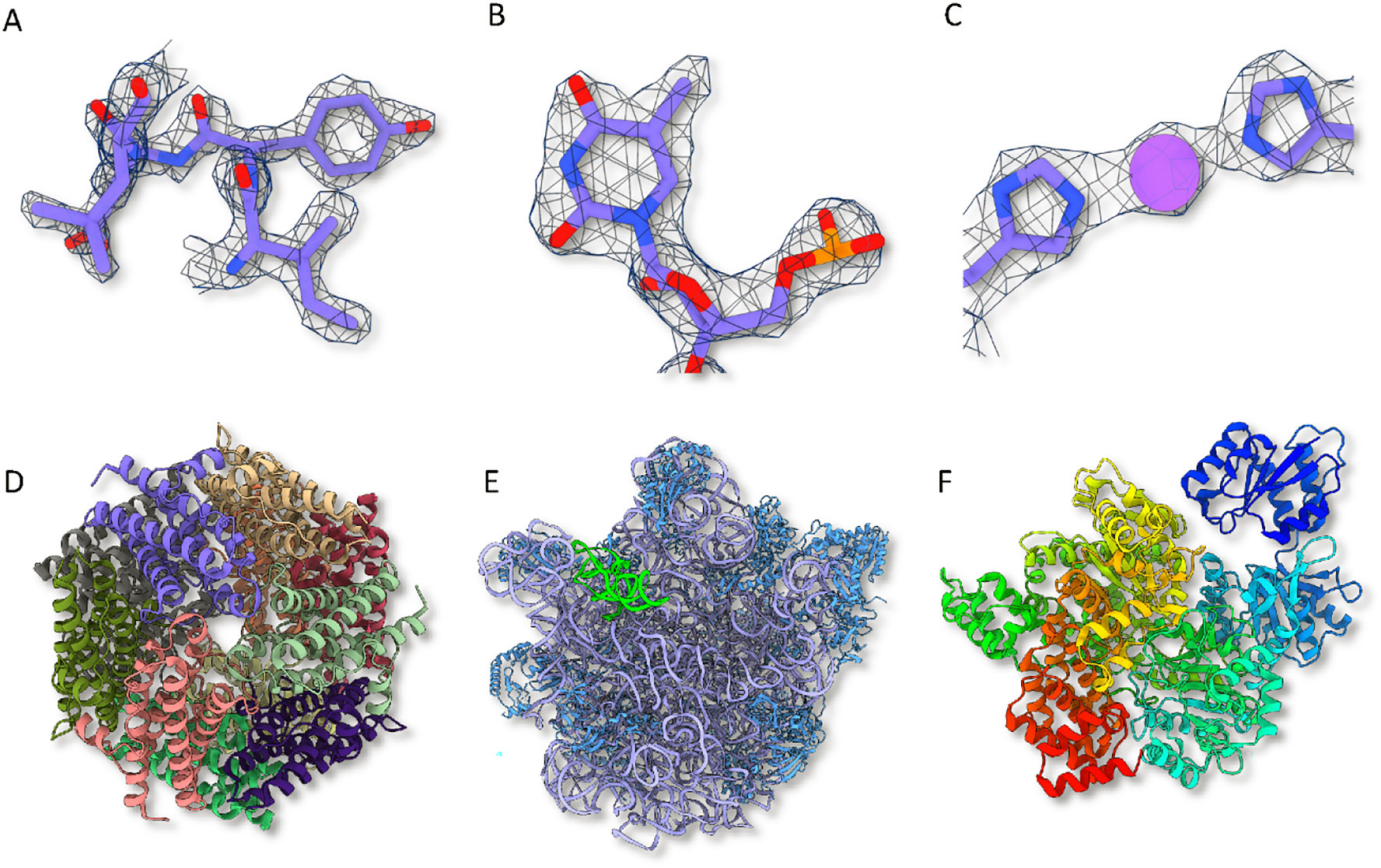
Representative density and structure. (A) Density for DPSL residues 74–79, (B) density for 5-methyluracil (5MU 1926) in the 50S ribosome. (C) Density for HmuS His538 and His1209, chelating a sodium ion. Ribbon structures of (D) DPSL colored by chain, (E) *Pseudomonas aeruginosa* 50S ribosome with an ‘E’ tRNA site colored green, 23S rRNA colored purple, and ribosomal large subunit proteins colored cyan, and (F) HmuS colored by domain.

**Figure 5:**
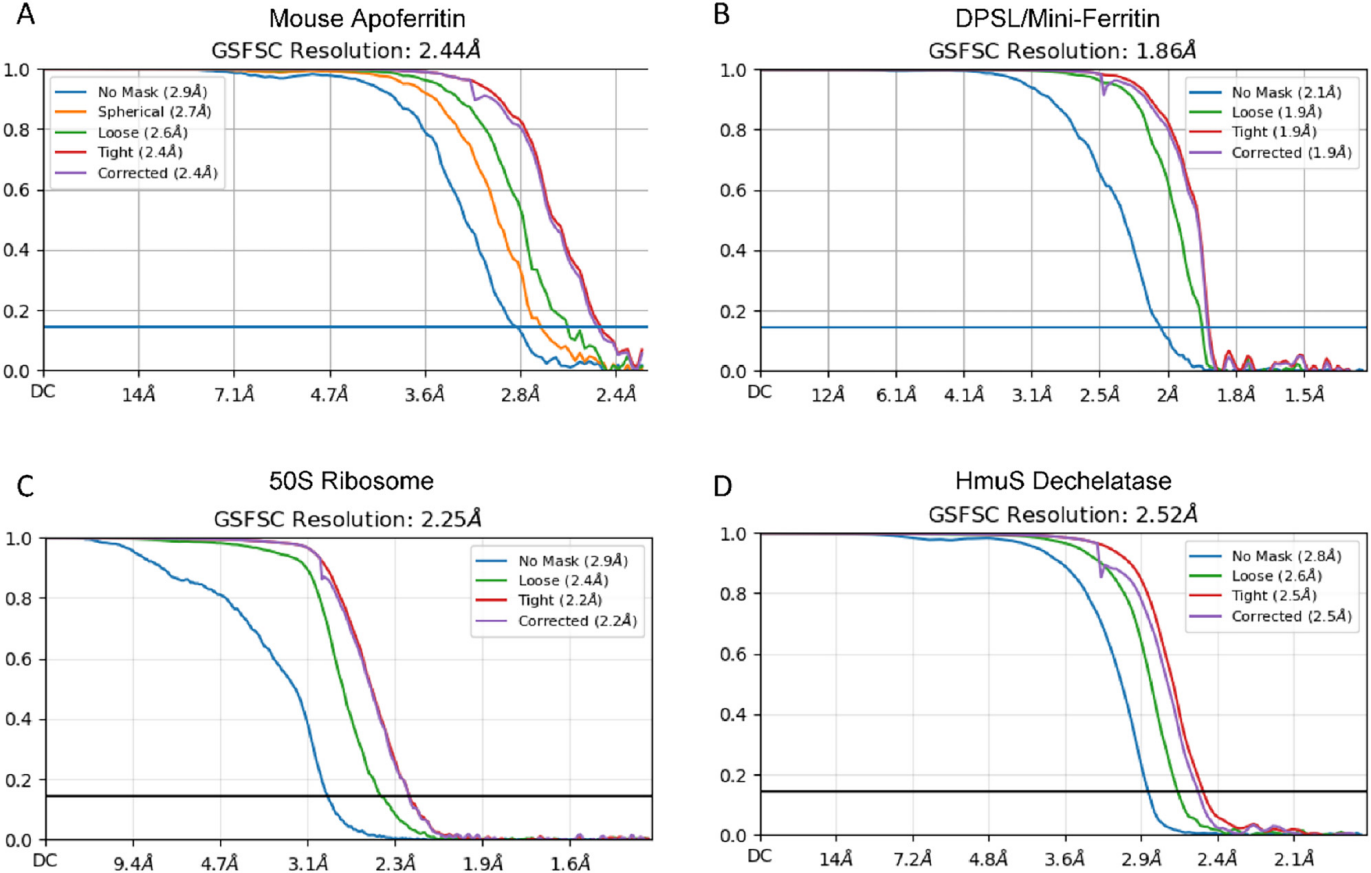
FSC curves. Gold standard FSC curves are shown for (A) mouse apoferritin, (B) DPSL/mini-ferritin, (C) the *Pseudomonas aeruginosa* 50S ribosome, and (D) HmuS dechelatase.

We previously described a new member of the mini-ferritin superfamily known variously as DPS-Like, DPSL and Thioferritin [29], [30], [31]. Like DPS, DPSL is a dodecamer. But unlike DPSL with its ferroxidase site on the interior surface of the hollow particle, it harbors a bacterioferritin like ferroxidase center that is present in the middle of the 4-helix bundle, suggesting an evolutionary link between the mini- and maxi-ferritins. We reasoned the size (258 kDa) and dodecameric symmetry of the particle might make this an appropriate first project, with a significant chance for success. We chose *Pyrococcus furiosus* DPSL specifically, because it had also been resistant to crystallization. Indeed, we ultimately resolved a 1.86 Å map of the unmineralized form of this particle (PDB ID 9CZ0, EMD-46055, Table 1). A representative micrograph, potential density and the fit of the model to the map are shown in Figures 3 and 4, and the gold standard FSC curve is presented in Figure 5. In conjunction with 3 additional Pf-DPSL structures, this work allowed us to elucidate the mechanism of iron mineralization in a mini-ferritin, and was recently published in JACS [32].

##### 3.4.1.2 *Psedomonas aeruginosa* 50S ribosome

A second early project focused on studies of the *P. aeruginosa* ribosome, with an ultimate goal of understanding the mechanism of ribosome hibernation [33], [34], [35], [36], [37]. In preliminary work, we utilized a complex ribosomal fraction isolated from exponential phase cells that contains dissociated 30S and 50S subunits in addition to assembled 70S ribosomes. While work with the 70S particles is still in progress, we have completed structures for the 50S particles in the mixture, resolving 8 high resolution structures in various assembly states and conformations. This includes a 2.25 Å resolution map for the dominant form (PDB ID 9YPH, EMD-73300, Table 1). A representative micrograph, potential density and the fit of the model to the map are shown in Figures 3 and 4, and the gold standard FSC curve is presented in Figure 5. A manuscript describing this work is nearing completion.

##### 3.4.1.3 Iron dechelatase

The DPSL and 50S projects had the respective advantages of high symmetry or large particle size with significant ordered RNA content. To assess our ability to work with smaller asymmetric particles, we pursued the structure of HmuS from *Bacteroides thetaiotaomicron* (*B. theta*). Heme is a major source of iron for the anerobic microbiome in the human gut. But because oxygen is needed for heme oxygenase to cleave heme and extract iron, it was unclear how these bacteria liberate iron from heme in the absence of oxygen. The structure of HmuS in complex with heme was determined at 2.6 Å (PDB 9D26, EMD-46483, Table 1). A representative micrograph, potential density and the fit of the model to the map are shown in Figures 3 and 4, and the gold standard FSC curve is presented in Figure 5. In conjunction with biochemical work, we learned that human intestinal microflora utilize HmuS as a dechelatase to directly extract iron from heme [38].

#### 3.4.2 Other applications

In addition to single particle work, we also see consistent interest in imaging cryo-preserved vesicles, liposomes, structurally heterogeneous viral particles and soft nanoparticles with applications in bio- and nano-technology. This includes work on the sequestration of polyfluoroalkyl substances (PFAS) in lipid bilayers and their effects on bilayer structure, i) extracellular vesicles, and ii) the development of drug targeting and delivery systems [39], [40]. For such work, we routinely collect a few tomograms in addition to individual micrographs. Both micrographs and tomograms routinely resolve the inner and out leaflets of the lipid bilayer, suggesting ∼2 nm resolution for these structures. In contrast, the resolution is significantly less when working with isolated organelles and small bacteria, and we avoid anything larger than this (Figures 4 and 5).

## 4 Discussion

### 4.1 Current limitations and future considerations for 200 kV Cryo-EM

The Montana State University Cryo-EM Core Facility is now serving academic and industrial users across the Northern Rocky Mountain region. While single particle analysis at the facility routinely provides sub 3 Å resolution structures, the work reported here is just one more example of this. Specifically while 2017–2019 saw only 3 reports of non-viral structures at better than 3.0 Å resolution [3], [4], [41], the flood gates began to open in 2020 with at least 17 more publications [7], [9], [26], [42], [43], [44], [45], [46], [47], [48], [49], [50], [51], [52], [53], [54], [55]. As of Feb 2026, a search of the PDB returns 439 non-icosahedral structures at better than 3.0 Å resolution, and 943 additional structures in the 3.0 to 3.5 Å bin. Thus, it is now well accepted that advanced 200 kV microscopes with direct electron detectors can routinely deliver high resolution (sub 3.5 Å) single particle structures. At the same time, however, a recent analysis by Danev et al. makes it clear that 200 kV microscopes remain heavily underutilized [56]. Regardless, despite these successes at 200 kV, the Talos Arctica described here has important limitations, especially relative to 300 kV microscopes. At the same time, there have also been significant new developments in 200 kV instruments. These limitations and relevant advances are now discussed.

#### 4.1.1 Fringe free imaging

First, while we routinely use a 5 × 5 multishot strategy to enhance our data collection rates, the two condenser lens system limits us to one shot per hole due to the width of the beam, whereas modern Krios and CRYO ARM 300 microscopes utilize more tightly focused beams and fringe free imaging to acquire multiple images per hole, significantly enhancing data collection rates. Notably, however 200 kV instruments like the more recent the Glacios2 and the CRYO ARM 200 also offer fringe free imaging. For these reasons, a fringe free imaging retrofit to the Arctica is under consideration. Also critical to a successful multishot strategy is the comma versus image shift alignment, which is done with SerialEM.

#### 4.1.2 Data collection rates

That said, data collection rates (images per unit time) are a complex mix of many experimental variables, including the properties of the EM grid, electron flux, total dose, camera speed, camera mode (hardware binning vs super resolution), data collection software, and more. In our case, we have optimized data collection rates (see methods) with SerialEM and SmartScope to typically give 6,000 to 10,000 movies per 24 h with R 1.2/1.3 grids. For context, the Pacific Northwest Cryo-EM Center (PNCC), which runs four 300 kV Krios microscopes, lists data collection rates on their Krios-1/Falcon 4i instrument at 2,000 to 10,000 movies per 24 h; and on their Krios/K3 instruments (Krios-2, Krios-3, Krios-4) at 2,000 to 18,000 per 24 h (https://pncc.labworks.org/instruments). The data collection rates on our Talos Artica are thus quite respectable, even in the absence of fringe free imaging.

#### 4.1.3 Sample thickness

A second limitation is sample thickness. As ice thickness grows, inelastic scattering and absorption result in signal loss at all energies. But because the inelastic cross section is greater at 200 kV than 300 kV, so too is signal loss as a function of sample thickness [57]. Thus, when working at 200 kV, it is even more important to optimize sample preparation to obtain thin ice than it is when working at 300 kV [7], [58], [59], [60], [61], [62]. When this is not possible, access to a 300 kV instrument is certainly beneficial.

#### 4.1.4 Energy filters

Energy filters are often employed to remove inelastically scattered electrons, allowing only zero-loss (elastically scattered) electrons to contribute to the image. This significantly improves the signal to noise ratio and image contrast, especially in thicker samples. Energy filters are thus an attractive technical option, especially for well-heeled facilities. But since the benefits of the energy filter are proportional to ice thickness, users that would benefit the most from the energy filter will also likely be inclined to seek out a 300 kV instrument (see above). Thus, given their cost, energy filters may not always be the most judicious choice for frugal core facilities running 200 kV instruments. In any event, when needed, unfiltered 200 kV data should provide strong supporting data for access proposals to 300 kV instruments at the National Cryo-EM Centers and their equivalents around the world.

#### 4.1.5 Cameras

Both the Gatan K3 and Falcon 4i cameras have the option to collect in “super-resolution” mode, as opposed to “hardware binned” counting mode. The relative merits of super-resolution data collection with a two condenser lens microscope such as the Arctica have been discussed by Feathers et al. [63]. While we were initially excited to utilize super-resolution mode for several high resolution data sets, we quickly became aware of the growing requirements to store such data, especially since running in super-resolution mode often contributed little to the final resolution. Thus, while we still occasionally utilize super-resolution mode, our default is to collect in hardware binned counting mode, which utilizes 1/4th the disk space, a significant savings when considering hundreds of terabytes or even petabytes of data.

While our Gatan K3 direct electron detector has performed well, the K3 and other high end cameras that are optimized for 300 kV instruments suffer from reduced detective quantum efficiency (DQE) [58] when used on 200 or 100 kV instruments. A recent development is the introduction of new detectors optimized for these lower voltages [64], [65]. Looking forward, there is currently a lot of work in this area, and hopefully these more sensitive cameras will become standard for 100 and 200 kV instruments in the near future. An additional improvement might be the development of cameras optimized for higher flux rates and larger active area, which could substantially increase data collection speeds.

#### 4.1.6 Cold FEGs and narrow gap pole pieces

The information limit of the microscope is dependent on the overall damping of the phase contrast transfer function (CTF) by the envelope function, which is composed of separate terms describing the spatial and temporal coherence of the beam, respectively. The 200 kV Talos Arctica described above utilizes an X-FEG, or thermally assisted field emission gun. Today, cold field emission guns (Cold FEGs or C-FEGs) are available on both the ThermoFisher Glacios 2 and the CRYO ARM 200 II (Cc = 1.8 mm) as well as the higher end 300 kV offerings [66], [67]. The cold FEGs have significantly reduced energy spreads (0.3 eV compared to 0.7 eV) and chromatic aberration, and thus show better temporal envelopes at high spatial frequencies. More recently, improved spatial envelopes for 100 and 200 kV microscopes has been achieved with the use of narrow gap pole pieces that reduce both spherical (Cs) and chromatic aberrations (Cc). While the spherical aberration in our Talos Arctica is 2.7 mm, that of the SP-TWIN objective lens in the 100 kV Tundra (ThermoFisher) is 1.6 mm, and for the 200 kV CRYO ARM 200 II with the optional narrow gap pole piece, Cs = 1.5 mm. Impressively, for the CRYO ARM 200 II, the combined effect of the C-FEG and narrow gap pole piece is an envelope function (and resolution) that rivals a 300 kV instrument [56]. Thus, it seems the usefulness of 200 kV instruments continues to increase. An important potential limitation of the narrow gap pole piece, however, is limited sample rotation, which might preclude tomography and micro-ED applications – a classic tradeoff between high resolution and experimental flexibility.

#### 4.1.7 Tomography and micro-ED

The Talos Arctica also allows the collection of dose symmetric tilt series (±60°) with SerialEM. However, 200 kV instruments are clearly suboptimal for tomographic studies with all but the thinnest of samples, especially since sample thickness increases as the stage is tilted. That said, for facilities lacking a 300 kV instrument, our experience is that it can still serve as a tool for cryo-ET screening and triage, allowing user to assess grid quality with smaller bacterial cells, subcellular organelles, viruses or nanoparticles before committing to higher-end, off-site instrumentation.

In addition to tomography, stage rotation on the Talos Arctica can be utilized to collect microcrystal electron diffraction (micro-ED) data with the K3 [68], [69]. However, while we see some interest in micro-ED, it is small relative to the number of users with non-crystalline samples. For facilities and staff with crystallographic experience, micro-ED might merit support, but for those lacking this expertise, referring users to outside facilities could be a more efficient alternative.

#### 4.1.8 The future of single particle analysis at 200 kV

Overall, at the time our instrumentation was ordered the 200 kV Talos Arctica and Gatan K3 camera were strong choices for single particle analysis. And in light of the technological advancements since then, the argument for 200 kV systems seems even stronger today, with a very bright future. This is ture not only for smaller sized Carnegie R1 universities (and their equivalents) for whom the acquisition and maintenance costs of a 300 kV system are prohibitive, but also for larger facilities where additional band width might be needed. In addition, they can also support cryo-ET screening and micro-ED if needed.

### 4.2 Users and access

The facility currently serves ∼25 academic users per year, and consistent with our mission as a regional facility for the Northern Rockies, roughly half are groups here at Montana State University, with the remainder distributed across Montana, Idaho and Eastern Washington. The average wait time for microscope access varies between a few days and 2 weeks, and is available on a first-come, first served basis. Importantly, as time allows, we also serve a small but active group of industrial users.

#### 4.2.1 User training

User training is accomplished in part with an introductory class in cryo-EM. In addition, three groups on campus have significant, self-sustaining expertise [6], [70], [71], [72], [73], [74], [75], [76], [77], [78], [79], [80], [81], [82], and each has a designated super user that in conjunction with our facility manager, are points of contact for additional assistance and training within that group. The super users are accomplished in basic microscope alignment, SerialEM and Smart Scope data collection, and are also allowed to load microscope grids. Inexperienced users from other laboratories are frequently paired with one of the three experienced cryo-EM groups. Otherwise, all Talos Arctica operations are done by, or under the direction of the facility manager in order to enhance instrument uptime and data quality.

The ultimate success of many facilities is directly tied to the success of its users. For this reason, we work with our users from start to finish to promote their success. Common considerations for beginning users, especially when working at 200 kV, is the need for thin ice and significant particle densities. We encourage users explore glow discharge parameters, longer blotting times with higher protein concentrations, and buffer parameters such as salt and surfactant concentrations [83], [84]. In addition, when on the fly processing indicates a potential structure, we encourage the collection of larger data sets. Because resolution frequently scales with the log of particle numbers, larger data sets will better support 3D variability and 3D classification to give multiple structures from a single data set. It is generally more cost-effective to capture large data sets at the earliest opportunity, than to collect additional data at a later time point.

On the computational side, we assign exposure groups at the earliest opportunity, allowing CTF refinement per exposure group. And once initial maps are in hand, we advise ResLog analysis to determine as quickly as possible whether the resolution is limited by particle numbers [85]. In addition, 3D variability and 3D classification should also be explored sooner rather than later. As processing nears completion, users are encouraged to evaluate the benefits of reference based motion correction, and to consider the interplay between defocus, resolution and box size (D + 2R, [86]), as they optimize box size and Fourier cropping parameters for the highest possible resolution.

### 4.3 Sustainability

Facile, local access greatly facilitates structural work. It allows quick iteration to refine sample preparation and data collection. It also provides valuable hands on experience and greatly contributes to the development of a local critical mass in cryo-EM, which in turn lowers the barrier for new users, and contributes to the success of the molecular life sciences research enterprise in general. But for many institutions, the cost to acquire and maintain even these 200 kV systems can still be a challenge. Further development of the user base may be necessary in order to balance the costs and relative benefits, and create a sustainable business model for the facility. For these reasons, the NSF MRI proposal that funded this work included a commitment to a tenure track faculty hire with expertise in cryo-EM, along with subsequent development of an introductory graduate level class (suitable for advanced undergrads) in cryo-EM. In addition, a supporting award from the Murdock Charitable Trust included mini-grant funds for new users, while partial support for the facility manager was secured by including the new Cryo-EM Core Facility in a phase 3 NIH CoBRE proposal from the University of Montana. Finally, we also market unused instrument time to industrial users, who are now also realizing the benefits of 200 kV instruments.

## 5 Conclusions

Smaller Carnegie R1 research intensive universities and institutes are well served by 200 kV microscopes for single particle work. These microscopes approach the capabilities of more expensive 300 kV instruments with their ability to produce high resolution single particle structures, and can do so at significant cost savings, contributing to sustainable core facilities. In addition, they provide screening capabilities for electron cryo-tomography (CET) and also serve those working with structurally heterogeneous viral particles, vesicles, liposomes, soft nanoparticles and other applications in bio- and nano-technology.
